# Modulation of Speech-in-Noise Comprehension Through Transcranial Current Stimulation With the Phase-Shifted Speech Envelope

**DOI:** 10.1109/TNSRE.2019.2939671

**Published:** 2019-11-18

**Authors:** Shabnam Kadir, Chrysoula Kaza, Hugo Weissbart, Tobias Reichenbach

**Affiliations:** 1 Department of BioengineeringImperial College LondonSouth Kensington Campus 4615 LondonSW7 2AZU.K.; 2 Centre for Neurotechnology, Imperial College LondonSouth Kensington Campus 4615 LondonSW7 2AZU.K.; 3 School of Engineering and Computer ScienceUniversity of Hertfordshire 3769 HatfieldAL10 9ABU.K.

**Keywords:** Transcranial current stimulation, speech envelope, speech-in-noise comprehension

## Abstract

Neural activity tracks the envelope of a speech signal at latencies from 50 ms to 300 ms. Modulating this neural tracking through transcranial alternating current stimulation influences speech comprehension. Two important variables that can affect this modulation are the latency and the phase of the stimulation with respect to the sound. While previous studies have found an influence of both variables on speech comprehension, the interaction between both has not yet been measured. We presented 17 subjects with speech in noise coupled with simultaneous transcranial alternating current stimulation. The currents were based on the envelope of the target speech but shifted by different phases, as well as by two temporal delays of 100 ms and 250 ms. We also employed various control stimulations, and assessed the signal-to-noise ratio at which the subject understood half of the speech. We found that, at both latencies, speech comprehension is modulated by the phase of the current stimulation. However, the form of the modulation differed between the two latencies. Phase and latency of neurostimulation have accordingly distinct influences on speech comprehension. The different effects at the latencies of 100 ms and 250 ms hint at distinct neural processes for speech processing.

## I. Introduction

Speech processing requires the brain to process information on the phonemic, syllabic, and word level in real time. Cortical activity tracks the broadband envelope of a speech signal, which can help to segment speech into distinct functional units [1]–[5]. In particular, magnetoencephalographic and electroencephalographic measurement of the cortical tracking have shown that tracking emerges at two distinct delays with respect to the audio signal [2], [6]. The early delay of around 100 ms thereby appears linked to the processing of lower-level acoustic features such as sound amplitude and phonemes [6], whereas the longer delay of about 250 ms may reflect the neural processing of more complex linguistic structures such as syntax and semantic information [5], [7].

Altering the cortical response to speech through transcranial current stimulation has been found to modulate the comprehension of speech in noisy backgrounds, evidencing its functional contribution to speech comprehension [8]–[10]. Two important parameters are therefore the latency and the phase between the alternating current applied and the speech envelope. Natural speech has a broadband envelope to which frequencies between 1 – 15 Hz contribute most [2], [4]. The envelope of natural speech is accordingly aperiodic: it is not dominated by a single frequency but by a broad range of frequencies. A phase shift of the envelope hence differs from a shift in time.

Previous studies on the modulation of speech comprehension through neurostimulation with the speech envelope measured either the influence of a phase shift or of a latency, but did not investigate the interaction between the two variables. Indeed, three studies employed speech that was artificially altered so that words occurred at a fixed rhythm of about 3 Hz or of 4 Hz [8]–[10]. Transcranial alternating current stimulation at the respective frequency and at different phase shifts or temporal shifts was then employed during sentence presentation, and was shown to influence neural activity and speech comprehension. However, due to the periodic nature of the artificially-altered speech signal, the phase shifts were equivalent to temporal delays, so both parameters could not be varied independently. A further study investigated natural speech in which the rhythm of words, and hence the speech envelope, had a broadband spectrum [8]. Simultaneous current stimulation with the speech envelope at different temporal delays relative to the audio signal was found to modulate speech comprehension, but phase shifts were not investigated.

Here we aimed to investigate whether phase and latency shifts of the current stimulation with respect to the audio signal modulate speech comprehension differently. Such a difference may be expected if the neural tracking of the speech envelope at short and at long latencies play distinct roles in speech processing, such as for lower-level acoustic and higher-level linguistic processing, respectively. The different roles of the short-latency and the long-latency component should emerge when stimulating the brain with a current mimicking the speech envelope, which is both delayed with respect to the audio signal by the latency of the corresponding neural component, and shifted by various different phases. In particular, one might expect a certain phase shift between the speech envelope and the audio signal to yield an enhancement of speech comprehension, and another phase to yield a diminishment. Together this could result in a cyclical modulation of speech comprehension according to the phase of the stimulation. If the neural tracking of the speech envelope at the short and the long latency play different roles in speech processing, then the cyclical modulation of speech comprehension may differ between the two latencies.

## II. Materials and Methods

### A. Participants

A total of 17 subjects (8 female, 9 male) participated in this study. All participants were native English speakers with normal hearing, no learning disabilities, and no history of migraines, neurological or mental health disorders. All participants were right-handed and were between 19 and 31 years of age (mean 23.4 years, standard deviation 3.7 years). They signed an informed consent form before the beginning of the experiment and were compensated for their participation. All experimental procedures were approved by the Imperial College Research Ethics Committee.

### B. Experimental Procedure

**Fig. 1.**
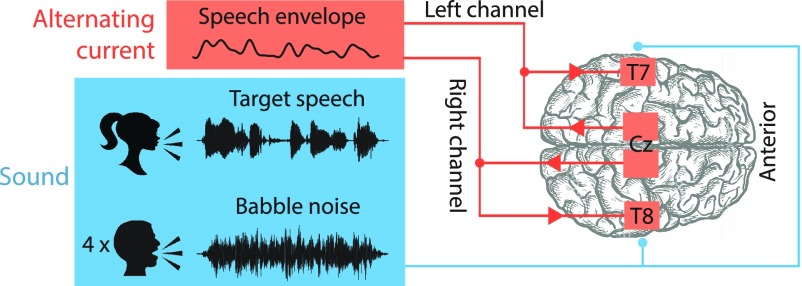
The experimental design. Participants listened to a female target voice that was presented in four-talker babble noise. The subjects were presented with the audio signal and simultaneously stimulated through a transcranial current that was based on the envelope of the target speech signal. The sentence reception threshold (SRT) at which the volunteers understood 50% of the target speech was determined behaviourally.

Subjects were presented with sentences spoken by a target female voice in background babble noise that was composed of four male speakers (Figure 1). The participants simultaneously received transcranial electrical stimulation. After hearing a sentence, the participant repeated what they heard and their answers were recorded and graded through automated speech-to-text conversion. The signal-to-noise ratio (SNR) of the sentence was varied through an adaptive procedure to estimate the SNR corresponding to the sentence reception threshold (SRT) at which the participant understood 50% of the target voice.

The SRT of each participant was measured for 16 different forms of applied transcranial current. As a control condition, we applied a sham stimulation that consisted of a brief initial current that lasted only 500 ms. We employed a DC anodal and a DC cathodal stimulation as additional controls, and stimulated with the envelope of an unrelated sentence as well. Six further conditions assessed the influence of stimulating with the speech envelope at a fixed delay of 100 ms but with (six) different phase shifts. In the remaining six conditions, we analogously employed a current that corresponded to the speech envelope shifted by the same six phases, but instead with a fixed temporal delay of 250 ms with respect to the speech signal.

The experiment was divided into two parts, each of which assessed the subject’s SRT for eight different forms of electric current stimulation. Participants undertook each of the two parts on different days. This ensured that the duration of the transcranial electrical stimulation did not exceed 20 minutes per day, in accordance with established safety protocols [11]. For each participant, the 16 different forms of transcranial current were randomized across the two sessions, and the order of their presentation within each session was randomized as well (eight conditions were assessed in each session). This resulted in a double-blind experiment where neither the participant nor the experimenter knew the order of the forms of the applied currents until both parts of the experiment had been concluded.

The volunteers were seated in a comfortable chair in a dimly-lit, anechoic chamber and electrodes were placed on their heads with an impedance of less than 10 $\text{k}\Omega $. The maximal current intensity to be used for stimulation was selected for each participant individually. To this end, a 3-Hz sinusoidal oscillation of five seconds in duration was presented. Its amplitude was gradually increased from $100~\mu \text{A}$ to a maximum of $1500~\mu \text{A}$ in steps of $100~\mu \text{A}$. After each increment, the participants were asked whether they felt any skin sensations or perceived a phosphene effect. When they answered in the affirmative for the first time, the procedure was stopped, and the current intensity of the penultimate step was set as the maximum value to be used throughout the whole experiment. The maximum current applied for the 17 participants lay in the range of 0.2 mA – 1.5 mA (mean 0.9 mA, standard deviation 0.42 mA). Animal and modelling studies suggest that the employed current, which was at most 1.5 mA and was applied through electrodes with a surface area of 35 cm^2^, was below the threshold required to trigger action potentials in single neurons [12]–[15]. The applied current could accordingly entrain cortical activity, but not evoke it.

In a short practice session prior to each experimental session, subjects were presented with the speech signals in multi-talker babble noise in order to gain familiarity with both the target voice and the form of the background noise. The sound level of the target voice was fixed at 75 dB SPL, both for the practice session and for the subsequent SRT assessments.

### C. Hardware Setup

Both the acoustic and the electrical stimuli were generated digitally on a PC (Windows 7 operating system). Both signals were converted to analogue waveforms through the USB-6212 BNC device (National Instruments, U.S.A.). The acoustic stimulus was then passed through a soundcard (Fireface 802, RME, Germany) and finally routed to insert earphones (ER-2, Etymotic Research, U.S.A.) that were placed in the subject’s ear canals. The signal for the electrical stimulation was fed to two stimulator devices (DC-Stimulator Plus, neuroConn, Germany). The devices converted the voltage signals to the desired current that stimulated the rubber electrodes attached to the subject’s head. We monitored the current signal through the neurostimulator devices as a control. The subject’s response was recorded with a microphone (Blue Snowball, BlueDesigns, U.S.A.).

### D. Electrode Montage

The two neurostimulation devices were each connected to two rubber scalp electrodes of 35 cm^2^ in surface area, one for stimulation and the other for the return of the current. To reduce the scalp impedance and achieve good contact, the sponge pads surrounding the rubber electrodes were moistened with a 0.9% saline solution before placing them on the head. The two stimulation electrodes were positioned over the auditory cortices at the positions T7 and T8 of the International 10–10 system. The two return electrodes were placed on either side of the Cz position; this symmetric setup induces relatively strong currents in both auditory cortices [8], [9], [16].

### E. Electrical Stimulation

The current stimulation with the speech envelope (env-tACS) employed the envelope of the target voice. The envelope of the speech signal was computed as the absolute value of the analytic representation obtained through the Hilbert transform of the speech signal. The envelope was subsequently low-pass filtered using a linear-phase filter with a cut-off frequency of 12 Hz.

**Fig. 2.**
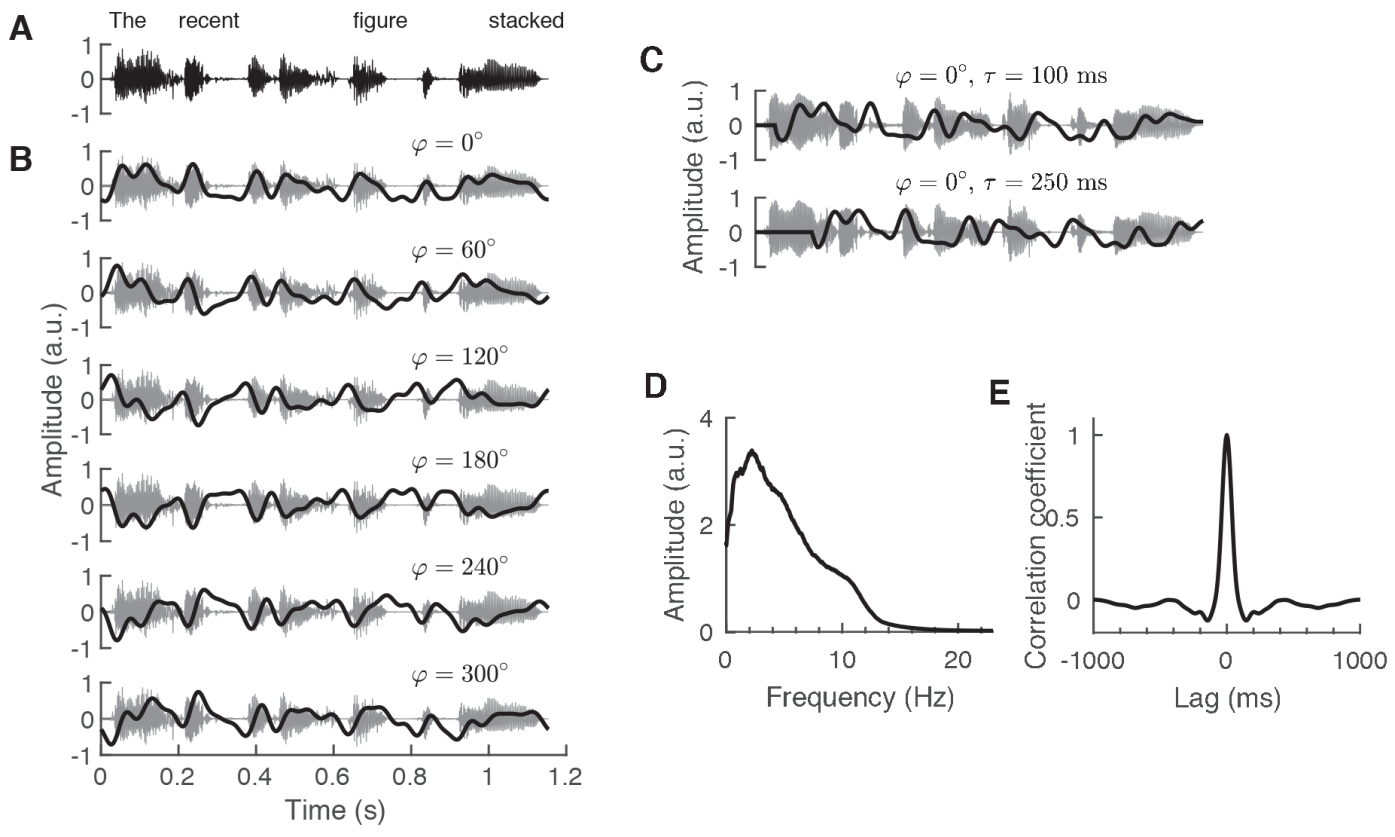
The audio and current signals. (A) The target sentences were semantically unpredictable. (B) The current stimulation was based on the envelope, but with the mean subtracted and and shifted by six different phases $\varphi $ (shown here without temporal delay). A phase shift of 180° corresponded to the inversion of the envelope. (C) The speech envelope with no phase shift but with a delay $\tau $ of 100 ms and 250 ms does not resemble the envelope obtained from either phase shift without delay. (D) The amplitude spectrum of the employed target speech envelopes was broadly distributed between 0 and 12 Hz. (E) The autocorrelation of the speech envelope was localized at 0 ms and was insignificant outside the range of −100 ms to 100 ms.

The phase-shifted version of the envelope $y(t)$ of the speech signal was computed through the analytical representation $z\left ({t }\right)=y\left ({t }\right)+iH[y(t)]$ of the envelope, a complexification of the real speech envelope obtained through its Hilbert transform $H[x(t)]$. Shifting this signal by a phase $\varphi $ is achieved through multiplying it by $e^{i\varphi }$. The phase-shifted envelope $y_{\varphi }\left ({t }\right)$ follows as the real part of this complex signal (Figure 2A, B):

\begin{equation*} y_{\varphi }\left ({t }\right)=Re\left [{ e^{i\varphi }z\left ({t }\right) }\right].\tag{1}\end{equation*}

To investigate how the env-tACS influences speech comprehension both at the early latency of 100 ms as well as the later delay of 250 ms, we shifted the obtained signals by both these delays relative to the audio signal. Hence, the current stimulation lagged behind the speech signal by either 100 ms or by 250 ms (Figure 2C). In addition, we subtracted the mean of each signal, so that the obtained waveforms alternated around zero. Potential offsets at the beginning and at the end of each stimulus from zero were smoothed by multiplying by a sine function centred at zero. The stimuli therefore began and ended at 0 mA.

### F. Generation of the Auditory Stimuli

We generated semantically unpredictable sentences using the Python Natural Language Toolkit [17]. The sentences respected syntactic rules but were not subject to any semantic restrictions and were therefore usually not meaningful, e.g. “The job fed the evening that slept”. Participants could thus not employ semantic information for word comprehension. All sentences contained a maximum of seven words, including five key words which were nouns, verbs or adjectives. Participants were tested on their ability to repeat the five key words correctly. Limiting the length of the sentences ensured that participants could retain all words in their short-term memory without difficulty. The sentences were converted to audio through the text-to-speech software TextAloud at a sampling frequency of 44,100 Hz.

To create the babble noise, sentences were synthesized with four different male voices and mixed together. Each of the target sentences was embedded in a seven second snippet of background babble noise. The sentence spoken by the target speaker which the participant had to identify and repeat, lasted 2.1± 0.3 seconds. The target voice onset was chosen to be 2.5 seconds after the babble noise onset.

### G. Adaptive Procedure to Estimate the SRT

The ability of the participants to understand the target voice in each of the 16 conditions was quantified through the sentence-reception-threshold (SRT), defined as the signal-to-noise-ratio (SNR) at which the subject understood 50% of the target words. We employed the weighted up-down method in which the SNR of the next sentence presentation depended on the performance of the subject in the current presentation: if the subject scored at least 50%, then the SNR for the next sentence presentation was reduced, whereas if the comprehension score was less than 50%, the SNR was increased for the subsequent sentence [18]. The initial SNR was 10 dB, and was changed by 3 dB for the first four reversals, that is, the first four changes in the direction of the SNR adaptation, and by a smaller increment of 1 dB in the following reversals. Around 25 sentences were employed to determine an SRT associated to a particular condition. The SRT was computed offline as the average of the SNRs of the last 10 sentence presentations. It therefore took about 15 minutes to measure one SRT. This procedure was employed to measure the SRT for each of the different conditions, that is, for the sham stimulation, the different env-tACS, as well as the DC+ and the DC- stimulation.

### H. Statistical Modelling and Analysis

We first sought to investigate the variation of the SRT with the phase of the stimulation at delays of 100 ms and 250 ms. Because of the circular nature of the phase, we employed circular statistics, and in particular utilized the Moore-Rayleigh test. This test is a modification of the Rayleigh test, applied to weighted vector data and seeks to establish whether there is significant variation with the vector angle [19]. It can thereby assess variations that have a period of 360°, or of 360° divided by an integer. Because we assessed the SRT at phases that differed by 60° or multiples thereof, the smallest period that we could consider was 120°, corresponding to a third of 360°. The larger periods at which we could assess variation were 180° (half of 360°) and 360° itself. As set out in Section III.D, a modulation at a period of either 180° and 120° evidences a nonlinear relation between the neurostimulation and speech comprehension. Because the test requires positive vector amplitudes, we subtracted the minimal overall SRT from each SRT. We adjusted for the resulting three comparisons per time lag through the Benjamini-Hochberg procedure.

We also quantified the statistical significance of the *differences* of the SRT’s dependence on the stimulation phase at the two latencies. To this end we computed, for each phase, the difference of the SRT at the long and at the short latency. The resulting differences at the various phases were then subjected to a Rayleigh-Moore test as well. This test assessed whether or not the phase dependence of the differences were significantly different from a uniform distribution, at the periods of 360°, 180° and 120°. The Benjamini-Hochberg procedure was employed to adjust for the three comparisons [20].

The Moore-Rayleigh test can only determine if there is significant modulation of the SRT by the phase at a single period, which in our case was either 360°, 180° or 120°. However, the actual dependence of the SRT on the phase may be a described as a linear combination of variations at several periods. Indeed, the Discrete Fourier Transform of a signal of $2N$ real numbers expresses this signal as a linear combination of a constant offset as well as of variations at $N$ different periods. These $N$ periods are fractions of the $2N$, the length of the signal. The largest period is $2N$, the second largest is $2N/2= N$, and the smallest is $2N/N=2$. In our measurement of the SRT’s variation with phase $\varphi $ we have six data points, such that $N=3$. The three periods are, when converted to phase, 360°, 180° and 120°.

The Discrete Fourier Transform employs complex coefficients. However, it can be recast into a variant, the Discrete Cosine Transform (DCT), that requires real coefficients only and which aids the interpretation of the results. For the SRT’s variation with phase $\varphi $ it takes the form

\begin{align*}&\hspace{-0.5pc}SRT\left ({\varphi }\right)=A_{0}+A_{1}cos\left ({\varphi -\psi _{1} }\right)+A_{2}cos\left ({2\varphi -\psi _{2} }\right) \\&\qquad\qquad\qquad\qquad\qquad\qquad\qquad\displaystyle {+A_{3}cos\left ({3\varphi -\psi _{3} }\right).} \tag{2}\end{align*}

The three amplitudes $A_{1}$, $A_{2}$ and $A_{3}$ denote hereby the strength of the variation at the periods of 360°, 180° and 120°, respectively. $\psi _{1}$, $\psi _{2}$, as well as $\psi _{3}$ are the phase offsets at those periods. The constant $A_{0}$ represents the mean of the SRTs.

We determined the three amplitudes $A_{1}$, $A_{2}$ and $A_{3}$ as well as the three phase offsets $\psi _{1}$, $\psi _{2}$, as well as $\psi _{3}$ in Equation (1) through the DCT. We then subjected the obtained amplitudes $A_{1}$, $A_{2}$ and $A_{3}$ to Least Absolute Shrinkage and Selection Operator (LASSO) regression with 10-fold cross-validation using the LARS algorithm [21]. The resulting statistical model was accordingly trained on 90% of the data and tested on the remaining 10% of data. The distinction between training and testing data ensured that the model did not overfit: although a statistical model could overfit the training data, such a model would produce a poor outcome when assessed on the testing data that were not used for training. The LASSO procedure therefore computed in a controlled way the least complex model that fitted the testing data best. The obtained sparse model allowed the assessment of which coefficients were nonzero, as well as their statistical significance, through a novel test developed by Tibshirani and others [21], [22].

Second, we also analysed the SRTs obtained from the sixteen different stimulation conditions through paired t-tests. In particular, we compared the sham stimulation to each of the remaining fifteen other stimulation types, and adjusted for multiple comparisons through the more conservative Benjamini-Hochberg-Yekutieli procedure, which makes no assumptions as to how the various comparisons may correlate with each other [23].

The statistical analysis was carried out at the population level.

## III. Results

### A. Relation Between Envelope Shift in Time and in Phase

We first verified that the envelopes of the speech signals that we utilized had a broad spectrum and that they were not dominated by a single frequency (Figure 2D). We found that the spectrum showed significant contributions in a broad range of frequencies from 1 – 12 Hz. Because the spectrum exhibited a peak at approximately 2 Hz, corresponding to a period of 500 ms, we wondered if this period dominated in the envelopes. We therefore computed the autocorrelation of the speech envelopes (Figure 2E). The autocorrelation showed a single peak at 0 ms, but no significant correlation at −500 ms, at 500 ms or another time lag. The speech signal was accordingly aperiodic.

**Fig. 3.**
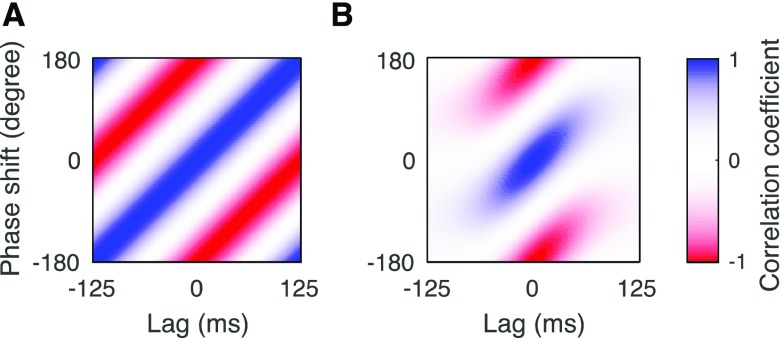
Relationship between envelope shifts in time and in phase. (A), The correlation of a sinusoidal oscillation at 4 Hz shifted by different delays and phases shows that the two shifts are dependent. In particular, a temporal lag can be compensated by a certain phase shift and vice versa. (B). When the speech envelope is shifted by different lags and phases, the obtained signals are only significantly correlated for latency shifts between −100 ms and 100 ms. In contrast, shifts by smaller or larger temporal delays lead to independent signals.

We further quantified the relation between shifts in phase and in time of the speech envelope. For a sinusoidally-varying signal, a shift in phase is indeed equivalent to a certain shift in time, and this becomes apparent when computing the correlation between such signals shifted by different phases and different delays (Figure 3A). In contrast, no such relation between phase shift and temporal delay exists for a broadband signal such as the envelope of the speech signals that we employed here. This was apparent from the phase-and time-shifted envelopes directly. If the envelope was periodic at 2 Hz, for instance, then a phase shift of 180° would be equivalent to a temporal shift of 250 ms. However, these two shifts yield envelopes that are visually very different (Figure 2B, C).

To further verify that the speech envelope shifted by a certain phase is independent from the envelope delayed by a certain lag, we computed the correlation of the envelope with different changes in phase and different temporal shifts (Figure 3B). We found that the signals were only significantly correlated if they did not differ by more than 100 ms in latency. In particular, an envelope with a delay of either less than −100 ms or more than 100 ms was uncorrelated from an envelope that had no time delay but was shifted by any phase. Because the delay of 250 ms and the delay of 100 ms were more than 100 ms apart, the phase-shifted envelopes at the delay of 100 ms were independent from those at a delay of 250 ms.

### B. Modulation of Speech Comprehension Through the Phase of Current Stimulation at Single Periods

The Moore-Rayleigh test revealed the statistical significance of the SRT’s phase dependencies at either of the three periods of 360°, 180°, and 120°. For the latency of 100 ms, we did not find significant changes at any period (360°, $p = 0.10$; 180°, $p = 0.12$; 120°, $p = 0.35$). At the latency of 250 ms, however, we obtained a significant modulation of the SRT by phase at the smallest period of 120° ($p = 7\text{e}$-4), but not at the longer periods of 360° and 180° ($p = 0.16$ respectively $p = 0.08$). Importantly, this variation emerged without adjusting the phase individually per subject, but instead showed consistent behaviour across subjects.

Moreover, we found that the phase dependence of the SRT at the latency of 100 ms differed significantly from that at 250 ms. In particular, the Moore-Rayleigh test on the difference of the SRTs at the two latencies revealed that the phase dependence of this difference was significantly different from a uniform distribution. Significant differences emerged at the period of 120° ($p = 2\text{e}$-4) and 360° ($p = 0.03$) but not at 180° ($p = 0.1$).

### C. Multiperiodic Dependence of Speech Comprehension on the Stimulation Phase

**Fig. 4.**
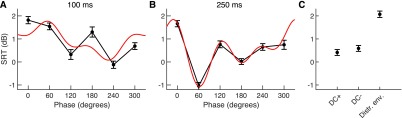
Modulation of the SRT through the transcranial current stimulation with respect to the sham stimulation (normalised to 0 dB). Results from the behavioural tests are shown as black dots; the error bars represent the standard error of the mean. The LASSO fit, involving only the significant terms, is shown as red line. (A) At a latency of 100 ms, a phase shift of 240° between the applied current and the envelope of the target voice gave the smallest SRT. The SRT at this phase was comparable to that of the sham stimulation. (B) When the latency of the stimulation was 250 ms, the phase shift of 60° produced an SRT that was about 1 dB below that of the sham condition, although the difference was not statistically significant. (C) Stimulation with a direct current yielded the same SRT as the sham stimulation, but a current at an unrelated envelope worsened the SRT significantly by 2 dB.

The phase dependence of the SRT can be a combination of variation at multiple different periods. Such a multiperiodic dependence can be aptly captured by the DCT (Equation 2, Figure 4). The amplitudes obtained from this transform describe the strength of variation at the different periods of 360°, 180° and 120°. Their statistical significance can be obtained from Least Absolute Shrinkage and Selection Operator (LASSO) regression in connection with cross-validation via the LARS algorithm (Methods) together with a novel statistical test developed by Tibshirani and others [21], [22].

**Fig. 5.**
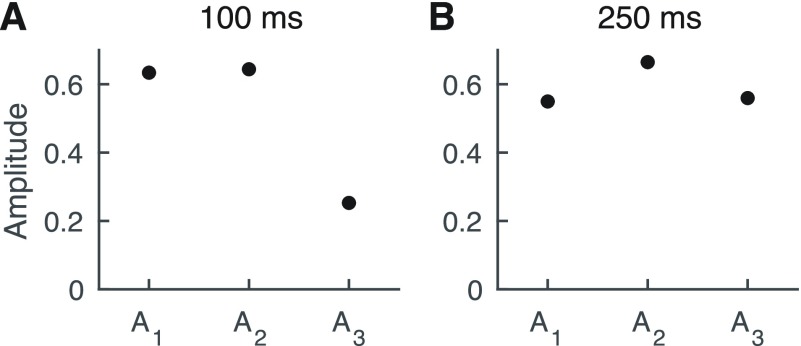
Multiperiodic dependence of the SRT on the stimulation phase. (A) At 100 ms, the amplitudes ${A}_{{1}}$ of the full period of 360° and ${A}_{{2}}$ of the period of 180° dominate over the amplitude ${A}_{{3}}$ of the shortest periods of 120°. (B) The modulation of the SRT at a delay of 250 ms of the current stimulation contains stronger modulation at shorter periods: the amplitude ${A}_{{2}}$ is the largest, and the amplitude ${A}_{{3}}$ is comparable to the amplitude of the component ${A}_{{1}}$.

At the early latency of 100 ms we found that the amplitude $A_{1}$ of the full period of 360° was similar to the amplitude $A_{2}$ of the period of 180°, and the amplitude $A_{3}$ of the period of 120° was nonzero as well (Figure 5A). Moreover, the analysis for statistical significance (using the novel method developed in [22]) revealed that both the amplitudes $A_{1}$ and $A_{3}$ were highly significant ($p < 1\text{e}$-4 for both), while the term $A_{2}$ was not ($p = 0.6$). We note that this behaviour emerged despite $A_{2}$ being larger than $A_{3}$. This shows that the SRT obtained from neurostimulation at a delay of 100 ms does indeed vary with phase, namely in a multiperiodic manner, with the two periods of 360° and 120°.

We also investigated the dependence of speech comprehension on the stimulation phase at the longer latency of 250 ms. We found that the amplitude $A_{1}$ as well as the amplitudes $A_{2}$ and $A_{3}$ were all of similar order, and were all highly statistically significant ($p < 1\text{e}$-4 for all three amplitudes; Figure 5B). This showed that the phase dependencies of the SRTs obtained at neurostimulation with a 250 ms delay was multiperiodic as well.

Regarding the phases for which neurostimulation yielded the best and worst speech comprehension, at the early latency, a phase shift of around 240° yielded the best SRT whereas a phase shift of 0° produced the worst speech comprehension (Figure 4A). The LASSO fit showed similarly a minimum at 254°, with an estimated SRT of −0.2 dB, and a maximum at 26°, yielding an SRT of 2.0 dB. At the longer latency of 250 ms, the best SRT emerged at a phase shift of 60°, and the worst at a phase of 0° (Figure 4B). The minimum in the LASSO fit occurred at 64°, with at SRT of −1.1 dB, and the maximum at 346°, with an estimate of 1.8 dB.

### D. Alignment by Best Phase for Each Time Lag

**Fig. 6.**
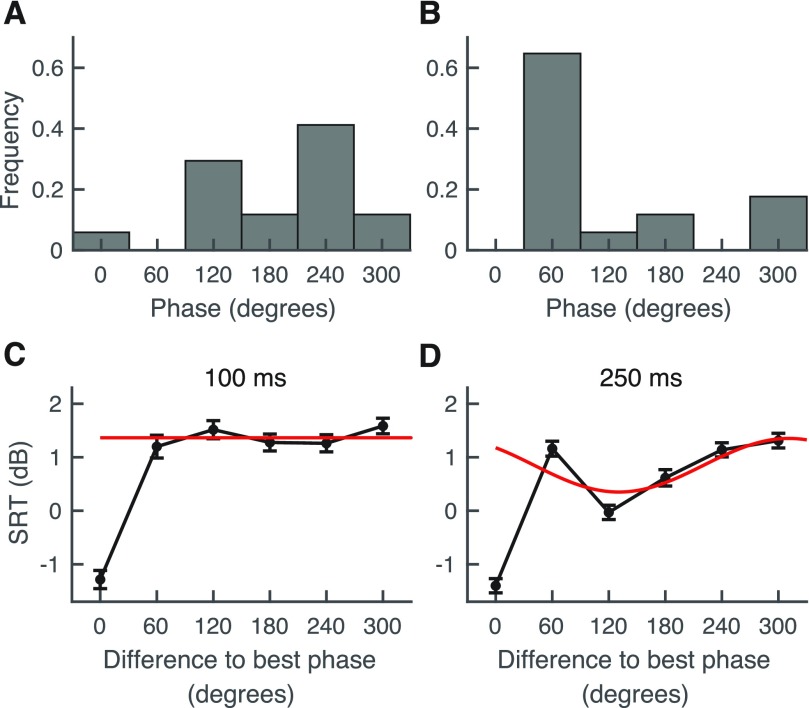
Variability of the best phase across subjects. The best phase denotes the phase at which a subject has its lowest SRT. (A) The distribution of the best phase at the latency of 100 ms appears relatively uniform. (B). The distribution of the best phase at 250 ms latency is clustered around the phase of 60°, and is significantly non-uniform. (C, D) The SRT s when aligned to the best phase per subject. After this alignment, the SRTs at the latency of 100 ms show no significant variation with phase (black dots and error bars: mean and standard error of the mean of the behavioural experiments; red line: LASSO fit with only the significant terms included). At 250 ms, there is significant variation in the SRT, but less than without alignment.

The phase of the env-tACS that yields the best or worst speech comprehension may vary from subject to subject. To explore such a putative inter-subject variation in the influence of the phase of the env-tACS on speech comprehension, we performed two types of analysis. First, we determined the best phase for each subject, that is, the phase that yielded the lowest SRTs for that subject. The distribution of the best phase, at the latency of 150 ms, was not significantly different from a uniform one (Rayleigh test, $p = 0.06$, Figure 6A). However, the distribution of the best phase at the longer delay of 250 ms deviates significantly from uniform (Rayleigh test, $p =0.007$, (Figure 6B). In particular, most participants have their best SRT at the phase of 60° at that latency.

Second, we aligned the phase of the neurostimulation with respect to the best phase for each subject (Figure 6 C, D). The best phase therefore corresponds to a phase difference of 0°, so that the SRT there is the lowest. We then analysed the phase-aligned SRTs for variation. We avoided analytic bias by omitting the SRT at 0° from any further statistical analysis [24]. Applying the LASSO procedure (LARS algorithm) for the remaining data points, at the latency of 100 ms, showed that there was no significant variation at either of the three periods ($A_{1}, p=0.2; A_{2}, p=0.6; A_{3}, p= 0.6$). For the latency of 250 ms, the amplitude $A_{1}$ of the first period was significant ($p = 0.0001$), but not those of the other two amplitudes ${(A}_{2}, p=0.1; A_{3}, p= 0.2)$. In contrast, as described above, without phase alignment, two out of three amplitudes were significant at the latency of 100 ms, and all three amplitudes were significant at 250 ms.

### E. Enhancement of Speech Comprehension and Comparison to Other Stimulation Types

The modulation of speech comprehension through env-tACS that we have shown results mostly in a worsening when compared to sham stimulation. However, our results also suggest that the env-tACS may be employed to improve speech-in-noise understanding. Indeed, stimulation at a latency of 250 ms and a phase of 60° yielded a SRT that was better than that of the sham stimulation, by 0.81 dB on average. However, the difference was not statistically significant ($p = 0.03$, paired t-test, $p = 0.29$ after Benjamini-Hochberg-Yekutieli adjustment for 15 multiple comparisons).

We aimed to establish how this SRT compared to other types of neurostimulation. We therefore measured the SRT in subjects when they were stimulated with a direct current. We employed the same electrode montage as for the env-tACS, and considered both the polarity with the cathode over the centre of the head (DC+), as well as with the anode over the centre of the head (DC-). The current intensity varied from subject to subject, and was the maximal one that had been determined for that particular individual before (Section II.B). We found that, compared to the sham stimulation, direct currents neither improved nor inhibited speech comprehension (Figure 4C).

If env-tACS with the speech envelope at an optimal subject-independent combination of phase and latency can improve speech comprehension, then stimulation with an unrelated speech envelope ought to act as a distractor and inhibit speech-in-noise comprehension. We accordingly assessed the SRT of subjects while we applied current stimulation with the envelope of an unrelated speech signal. This type of env-tACS, worsened the SRT significantly by 2 dB as compared to sham stimulation ($p = 8\text{e}$-5, paired t-test, $p = 0.002$ after Benjamini-Hochberg-Yekutieli adjustment for 15 multiple comparisons, Figure 4C). The worsening of speech comprehension was presumably due to the disruption of the cortical entrainment to the envelope of the target sentence that the subject was trying to listen to.

### F. Nonlinear Modulation of Speech Comprehension

We wondered if the dependence of the speech comprehension on the phase of the stimulation could be explained by a linear model. In particular, if speech comprehension depends linearly on neurostimulation signal $y_{\varphi }\left ({t }\right)$, then linear response theory states that the SRT can be expressed as

\begin{equation*} SRT(\varphi)=\int _{t_{a}}^{t_{b}} {\chi (t-t')y_{\varphi }\left ({t' }\right)dt'},\tag{3}\end{equation*}

with the susceptibility $\chi (t)$ [25], [26]. The time points $t_{a}$ and $t_{b}$ denote the beginning and the end of the speech presentation, respectively, that coincide with the beginning and the end of the neurostimulation. It easily follows from (1) and (3) that the dependence of the sentence reception threshold SRT on the phase shift $\varphi $ is sinusoidal:

\begin{equation*} SRT(\varphi)=A_{1}cos(\varphi -\psi _{1}),\tag{4}\end{equation*}

with an amplitude $A_{1}$ and a phase offset $\psi _{1}$ that are obtained from the susceptibility and the speech envelope. In contrast, it follows that any deviation from a sinusoidal dependence evidences a nonlinearity in the dependence of the speech comprehension on the neurostimulation. Such a nonlinearity could result from a nonlinear dependence of cortical activity on the applied current, from a nonlinear relation between speech comprehension and cortical activity, or from both.

The dependence of the SRT on the phase hints at a nonlinear response. Because a purely linear response would give rise to a sinusoidal variation at a period of 360°, a single minimum and a single maximum should emerge, and they should be 180° apart. However, at both latencies of 100 ms and 250 ms our data show two local minima and two maxima, and they differ by much less than 180°. Moreover, for both latencies, the two minima are 120° apart. This suggests a contribution at a period of a third of that of the linear response, corresponding to a nonlinear response.

The multiperiodic nature of the SRT’s phase dependence that we described above evidenced the presence of a nonlinear response (Equation 2). In particular, the significant component at the period of 120° at the latency of 100 ms, as well as the significant components at the periods of 180° and 120° at the 250 ms delay, constituted nonlinear responses. Nonlinearities in the modulation of speech comprehension through neurostimulation thus emerged both at the early and the late latency.

## IV. Discussion

Taken together, our results show that the modulation of speech comprehension through the phase of the stimulation differs between the short latency of 100 ms and the long latency of 250 ms. In particular, the phases of the stimulation that increases/decreases speech comprehension the most are different for each latency. In line with our hypothesis, phase shifts and temporal shifts of the current stimulation with respect to the envelope of broadband speech therefore modulate speech comprehension in different ways.

The different modulation of speech comprehension at the two different latencies may reflect different roles that both latencies play in speech processing. The early component of neural entrainment reflects processing of relatively low-level acoustic features such as onset detections [2], [27]. The later component has been shown to correlate more with higher-level processing such as of semantic information [7]. A further understanding of the neural mechanisms of speech processing may hence lead to better types of current stimulation for speech enhancement, and current stimulation may in turn provide a tool to probe neural mechanisms of speech comprehension.

Our results also show that current stimulation with the phase-shifted envelope of speech allows the modulation of speech comprehension in a way that is consistent across participants. In particular, we did not employ a subject-dependent reference of the phase, such as an optimal stimulation phase for each particular subject. In contrast, when we aligned the phase per subject with respect to the phase that gave the best speech comprehension for that subject, the modulation of speech comprehension became more noisy and less significant (Figure 6C, D). This suggests that the phases at which neurostimulation improves respectively deteriorates speech comprehension are relatively consistent across subjects. Indeed, at the latency of 250 ms, we observed that the preferred phases per subject were non-uniformly distributed and clustered around 60°.

This accords with the universality of the neural entrainment to the speech envelope, the source and timing of which is very similar between different individuals [2], [3], [28]. Previous studies on the effect of env-tACS on speech comprehension have, however, obtained results that strongly differed from subject to subject [8]–[10]. These studies have either employed artificially-altered speech that was made to follow a single rhythm, or neurostimulation with an envelope that did not account for a possible phase difference between the stimulation and the envelope of the acoustic waveform. Because our approach allows to segregate phase and latency of the neurostimulation as compared to the acoustic signal, it will be useful in further studies to clarify the origin and nature of inter-subject variability in the modulation of speech comprehension through non-invasive current stimulation.

Regarding the modulation of speech comprehension by the phase of the stimulation, we have employed two different tests for statistical significance that have yielded somewhat different results. In particular, the Moore-Rayleigh test revealed significant variation only for the delay of 250 ms, and only for the period of 120°. The multiperiodic analysis of the SRT’s dependence on the phase of the neurostimulation, however, revealed significant modulation at almost all periods, except for the one of 120° at the early delay of 100 ms. This evidences the greater proficiency of the statistical test based on the LASSO regression. The latter can indeed assess significance of more complex models that contain several components such as variation at different periods. The Moore-Rayleigh test, in contrast, assesses the statistical significance of the variation at each period by itself, without taking the variation at other periods into account. It therefore represents a more conservative but less complete test than the one based on the multiperiodic modeling.

We have also shown that transcranial current stimulation at both the early and the late latency modulate speech comprehension in a nonlinear manner. This finding was based on the multiperiodic modeling of the SRT’s phase dependence. The multiperiodic model that we employed represented the DCT of the data, which is similar to the discrete Fourier transform (DFT) [29]. Because both the DCT and the DFT decompose the dependence of one variable on a second variable into a linear combination of oscillatory components, these methods are particular suited to the analysis of circular data such as the phase dependence of the SRT that we have analyzed here [30]. However, as opposed to the DFT, the DCT uses only real numbers, making it well suited for the analysis of real-valued circular data. Moreover, following the well-established linear response theory, only the $A_{1}$ term in equation (2) of the DCT captures the entire linear response of the system, whereas the other terms reflect nonlinear behaviour (Section III.F) [25], [26]. We would like to emphasize that, although the nonlinear contributions could be expressed through many different types of functions, any such model will necessarily recapitulate our finding that the dependence of the SRT on the phase contains nonlinear contributions.

The nonlinear modulation of speech comprehension by neurostimulation that we found can reflect a number of nonlinear processes in the brain. Cortical activity arises indeed from an intrinsically nonlinear system: single neurons respond in a highly nonlinear fashion to their input, and networks of neurons can therefore exhibit all aspects of nonlinear dynamics, from multi-stability to limit-cycle oscillations and chaos [31], [32]. The modulation of cortical entrainment through the current stimulation is therefore likely nonlinear, as is the modulation of speech comprehension through the neural tracking of the speech envelope. Further investigation of the nature of this remarkable nonlinearity may employ computational modelling of neural network dynamics, such as through a recently proposed spiking neural network for speech encoding, and may reveal how current stimulation can be optimized to modulate speech-in-noise comprehension [33].

We assessed the role of neurostimulation on the comprehension of speech in multi-talker babble noise. Understanding speech in noise is indeed one of the most challenging daily tasks for the human auditory system, and people with hearing impairments complain mostly about difficulty with understanding a speaker when others talk at the same time [34], [35]. Neurostimulation may influence speech comprehension through modulating the separability of the speech signal from the background noise or through a more intrinsic role for speech processing such as phoneme detection. A recent study on degraded speech with concurrent env-tACS, but in the absence of background noise, has shown that current stimulation modulates speech comprehension in that condition as well [8]. Future studies may employ the method of phase- and latency-controlled env-tACS that we introduced here to probe its influence on speech processing in the absence of background noise.

Although we showed that neurostimulation with the speech envelope modulates the comprehension of speech in noise, the current stimulation resulted mostly in a worsening of speech comprehension when compared to sham stimulation. At a few phase shifts and latencies, however, did the neurostimulation yield speech comprehension that was comparable to that under sham stimulation. Although modest improvements up to 0.81dB were seen for some parameter values, this was not significant. This is, however, in line with previous studies on this topic that did not find an improvement either [8], [9]. An important further line of investigation will be to determine if the shape of the applied current can be optimized to improve speech-in-noise comprehension. Such an optimized current that may, for instance, be designed to enhance the cortical tracking of higher-level acoustic features such as phonemes. Moreover, further investigations are needed to establish the temporal duration over which such currents can be safely delivered to a human participant. Apart from potential applications in aiding speech-in-noise comprehension, the combined acoustic and electric stimulation opens up possibilities for a non-invasive treatment of neurological disorders that may involve an impairment with cortical entrainment such as developmental dyslexia and schizophrenia [36]–[42].
